# Longitudinal Changes in the Structure of Speech Categorization Across School Age Years: Children Become More Gradient and More Consistent

**DOI:** 10.1111/desc.70085

**Published:** 2025-11-03

**Authors:** Ethan Kutlu, Hyoju Kim, Bob McMurray

**Affiliations:** ^1^ Department of Communication Sciences & Disorders, Department of Psychological & Brain Sciences, Department of Linguistics University of Iowa Iowa City USA; ^2^ Department of Psychological & Brain Sciences University of Iowa Iowa City USA; ^3^ Department of Psychological & Brain Sciences, Department of Communication Sciences & Disorders, Department of Otolaryngology – Head and Neck Surgery, Department of Linguistics University of Iowa Iowa City USA

## Abstract

A critical aspect of spoken language development is learning to categorize the sounds of the child's language(s). This process was thought to develop early during infancy to set the stage for the later development of higher‐level aspects of language (e.g., vocabulary, syntax). However, many recent studies have shown that speech categorization continues to develop through adolescence. This work, which uses more sophisticated forced‐choice tasks and stimuli that span speech continua (e.g., /b/ to /p/ in small steps) suggests that children show increasingly sharp category boundaries over development. However, this contrasts with the consensus view in adult speech perception that speech categories are graded and this may help listeners be more flexible. Here, our longitudinal study revisited these results using a new Visual Analogue Scaling (VAS) task, which employs a continuous rating scale to potentially unpack the underlying cause of a shallower or more gradient boundary. We tested 225 school‐aged children (in grades 1–3) over 4 years (through grades 4–6). A Bayesian Hierarchical psychometric model was fit to extract the Slope of the categorization function and Response Consistency (the trial‐by‐trial variance). Results show that as children age, they show shallower (more gradient) slopes. It also highlighted a new variable, suggesting that older children exhibit greater trial‐by‐trial categorization consistency. These findings suggest that sound categorization continues to develop through early school age. These findings also suggest that children become increasingly sensitive to fine‐grained gradient detail in the signal. The dramatic changes in consistency represent a challenge to current theoretical models that focus primarily on underlying category representation.

**Summary:**

- Speech categorization continues to develop through the school years, refining children's ability to categorize speech sounds more precisely and flexibly.
- A longitudinal study tested 225 school‐aged children using a Visual Analogue Scaling (VAS) task and Bayesian Hierarchical psychometric modeling.
- With age, children showed shallower (more gradient) categorization slopes and greater trial‐by‐trial response consistency.
- Findings challenge theoretical models, revealing continued development of speech categorization and increasing sensitivity to fine‐grained gradient detail in the signal.

## 1 Introduction

Speech perception is challenging. Listeners must parse a variable and continuous acoustic signal in which the form of even basic sounds like /b/ and /p/ is affected by the identity of the talker, their speaking rate, neighboring phonemes, and many other variables (Chodroff and Wilson 2017; Gay 1978; Hillenbrand et al. 1995; McMurray and Jongman 2011; Miller et al. 1986a, 1986b; Theodore et al. 2009; Viswanathan et al. 2014). To illustrate, sounds like /b/ and /p/ are distinguished in part by continuous acoustic cues like Voice Onset Time (VOT), the time difference between the release of the articulators and the onset of voicing. In American English, /p/ has a longer VOT near 50 ms, and /b/ has a shorter one near 10 ms. However, the VOTs associated with any sound can shift. For instance, a slower speaking rate can lengthen VOTs (Miller et al. 1986b), and individual talkers may have their own characteristic VOTs (Allen and Miller 2001). Thus, depending on these factors, the same VOT might be consistent with either /b/ or /p/.

Development compounds this problem. Different languages carve up the acoustic space in different ways. Spanish, for example, has voicing categories centered at −60 and 10 ms, while Thai has three categories. Consequently, children must learn the categories of their language(s)—the long‐term phonological representations, while also learning the real‐time skills that allow them to compensate for this inherent variability.

This problem was thought to be solved early in development (Kuhl 2004; Werker and Hensch 2015). Infants as young as 6 months can discriminate most speech contrasts used across the world's language(s) (Kuhl 2004; Kuhl et al. 2006; Narayan et al. 2010; Werker and Tees 2005). However, this openness appears to narrow through about the first 18 months (for most contrasts). During this period, infants’ ability to categorize speech sounds becomes more refined and tailored to the language that they are exposed to regularly (Kuhl 2004; Kuhl et al. 2006; Narayan et al. 2010; Werker and Tees 2005)—they lose the ability to discriminate sounds of other languages, and enhance their ability to discriminate native language contrasts (Eilers et al. 1977; Galle and McMurray 2014; Kuhl et al. 2006; Narayan et al. 2010; Tsuji and Cristia 2014). This empirical phenomenon has been termed *perceptual narrowing* (Maurer and Werker 2014), reflecting the loss of discrimination for non‐native contrasts. Such narrowing is a robust phenomenon that has been shown across multiple speech contrasts in various studies, though recently it has been reframed as *attunement* (Werker 2024).

However, developing children must not only learn the set of categories that are required for their native language(s). They must also acquire categories that have the appropriate “structure.” That is, they must learn how much variability in phonetic cues to accept, and how gradient or discrete those categories are. This process is less well‐studied, and three basic—but critical—questions remain unanswered. First is the developmental timecourse: over what ages does the structure of speech categories evolve? Second is the nature of the development: are children moving toward more discrete categories, or something more gradient? Finally, are there other perceptual dimensions—beyond the nature of the categories—that co‐develop to enable accurate speech perception?

### 1.1 Developmental Timecourse

With respect to the developmental timecourse, a number of studies with older children suggests that categories continue to be refined through childhood or later (Hazan and Barrett 2000; Nittrouer 2002; Slawinski and Fitzgerald 1998)—speech perception continues to develop through at least age 12. These studies used forced‐choice categorization tasks with various speech continua (what we term Forced‐Choice + Continua or *FC+C* tasks). In these tasks, participants hear tokens from a speech continuum (e.g., from /b/ to /p/) and then explicitly identify the speech sound. The dominant story across multiple studies in childhood is that the slope of children's identification function gets steeper with development—they become more categorical or more precise. Similarly, studies of children with dyslexia, children with developmental language disorders, and linguistically diverse children often show shallower slopes than typically developing children (Robertson et al. 2009; Werker and Tees 1987), reinforcing the view that sharper (more categorical) identification profiles are developmentally desirable. However, this development has not been assessed longitudinally, leaving the precise developmental window fairly open.

### 1.2 The Developmental Target: Categorical or Gradient Categories

The nature of the development has far less clarity. Here, there appears to be a contrast between the literature on the development of speech perception and what is known about the mature system. At face value, the aforementioned studies on older children and clinical populations using FC + C tasks suggest a system that is developing toward a more discrete or categorical representation of speech: older children who have steeper categorization slopes (more categorical), and in clinical populations, whose identification functions are shallower or more gradient. This is consistent with categorical perception (CP) and related accounts of adult speech perception (Liberman et al. 1957; see McMurray 2023 for a review) which stress that the goal of the system is to discard or ignore unnecessary differences in the fine‐grained phonetic instantiation of a category (e.g., whether the VOT is 10 or 15 ms), to only attend to differences that are phonemically contrastive. This is also a reasonable extension of perceptual narrowing accounts to this question, as again the system is developing toward one in which there is less sensitivity to less relevant auditory contrasts (though it is important to note that perceptual narrowing was largely posited as a description of the process of identifying native language categories, not determining their structure).

Under these views, listeners are attempting to ignore input variation within a category (e.g., non‐native discriminations). If successful, it would not matter whether the VOT is 0 ms (a good /b/) or 15 ms (less good)—these are both /b/’s. In contrast, a shallow or more gradient slope implies that some tokens which lie on the same side of a boundary are nevertheless treated differently. Thus, a shallow or gradient slope (i.e., a less steep slope) in a population like children with dyslexia has been linked to incomplete narrowing or incomplete CP (Serniclaes et al. 2004).

However, the consensus view in adult speech categorization suggests an alternative developmental target: children may be developing toward more gradient categories. Three arguments support this.

First, studies of adults offer a wealth of evidence that speech categories are gradient, reflecting fine‐grained within‐category differences. In priming, eye‐tracking, goodness rating, and ERP paradigms, VOTs (e.g.) near the center of the category (e.g., a 50 ms /p/) more robustly activate that category than those near the edges (20 or 100 ms), even though those edge tokens are consistently perceived as the same category (Andruski et al. 1994; Kapnoula et al. 2017; Massaro and Cohen 1983; McMurray et al. 2002a; McMurray, Tanenhaus, et al. 2009; Miller and Volaitis 1989; Sarrett et al. 2020; Toscano and McMurray 2010). This implies that listeners are not just tracking the category, but the relative goodness of the input, or in some cases, even the raw VOT.

Building on this, theoretical accounts suggest that this gradiency may enable listeners to be more flexible—a shallower slope may be beneficial. This is supported by empirical and theoretical work demonstrating that gradient representations help listeners in handling ambiguity, integrating information, recovering from misperceptions, and learning and adapting to speech variations (Bent et al. 2009; Gwilliams et al. 2018; Kutlu et al. 2024; McMurray et al. 2008, 2002, 2009; McMurray and Jongman 2011; McQueen 1996). This body of work with adults challenges the notion that children are attempting to achieve discrete categorization. Instead, they may be attempting to form gradient categories that are precisely tuned to the need for flexibility and variation in the language learning environment.

Second, this gradiency in adult speech categories is often presumed to derive from the same statistical learning mechanisms by which infants attune to their native language speech categories (Clayards et al. 2008; Farris‐Trimble and McMurray 2013; Kleinschmidt and Jaeger 2015; Pierrehumbert 2003; Theodore and Monto 2019). That is, many speech cues like VOT naturally form Gaussian‐like distributions across utterances in the language learning environment. If infants are using statistical learning mechanisms to acquire categories, these will naturally reflect this gradient distribution.

Third, a handful of developmental studies have attempted to address this issue directly. In infancy, two studies have explicitly found evidence that young infants are sensitive to within‐category differences (McMurray et al. 2008; Miller and Eimas 1996). However, both examined discrimination of pairs of VOTs (a good /p/ vs. a poorer one), making it impossible to fully characterize the structure of the category, and both examined only a single age. Similarly, Galle and McMurray (2014) pool results across studies to show some evidence for increasing gradiency during late infancy, but this result is limited to voicing, and robust meta‐analytic statistics were not possible given the nature of that review.

A single study of older children suggests increasing gradiency. McMurray et al. (2018) tested 7‐ to 18‐year‐olds on voicing and fricative place continua using the Visual World Paradigm. Although mouse‐click responses (forced choice) showed a steeper identification slope with age—suggesting more CP—eye movement data revealed that older children were actually more sensitive to fine‐grained acoustic detail. This indicates development toward a more gradient representation of speech. However, it remains unclear how to reconcile this increasing gradiency with the concurrent steepening of the identification slope.

Thus, there is a mismatch between the overwhelming empirical evidence for steepening category structure from forced‐choice tasks in childhood, and the strong theoretical rationale from adults for a more gradient target to development (with limited empirical support).

### 1.3 Measurement in Speech Categorization

In attempting to resolve this paradox, a closer consideration of the FC+C paradigm is warranted. How can the same psychometric response (a shallower slope) be consistent with both poorer (noisier) and better (more gradient) categorization? Part of the issue is that traditional forced‐choice tasks have an underlying ambiguity that makes it impossible to disentangle what a shallow categorization slope means (Apfelbaum et al. 2022).

To understand this issue, it helps to consider a few “case studies”—situations that illustrate the limitations of the FC + C tasks. In forced‐choice tasks, even if the underlying representation were gradient, a listener could still look categorical if they always chose the most likely response at each step along the continuum. Alternatively, if a listener showed a gradient response, they could be underlyingly gradient and attempting to match that underlying function by probabilistically selecting each option, or they could be categorical but with noise in the system, leading tokens near the boundary to be misperceived as the wrong category from trial to trial (Apfelbaum et al. 2022; Kutlu et al. 2022). Thus, either a categorical or gradient underlying category structure can give rise to a steep or shallow slope, depending on the assumptions that link the underlying structure to the responses in the task.

Recent work suggests that Visual Analog Scale tasks (VAS) can tease these factors apart (see Apfelbaum et al. 2022 for a review). Like the FC + C paradigm, listeners respond to a token from a speech continuum. However, instead of a discrete choice (i.e., 0 or 1), listeners respond on a continuous (analog) scale to indicate where the sound falls between endpoints. For instance, if a listener hears tokens from a *time/dime* continuum, the scale has an image of *time* on the left and a *dime* on the right, and listeners can choose whatever location corresponds to their percept.

This simple change—from a discrete to a continuous response mode—can overcome the interpretative ambiguity of the AFC task by considering the trial‐by‐trial variability (categorization consistency) alongside the slope of the average function.

For example, a listener with categorical representations but noisy encoding will show an average VAS function that is gradient, but high trial‐by‐trial variability around that mean (low consistency), as responses jump between endpoints. In contrast, a truly gradient representation yields a similar mean function but with low response variability, as responses cluster around the mean. Listeners likely fall between these extremes, varying in both gradiency (slope) and consistency (response variability). This distinction can clarify developmental changes in speech categorization.

Two recent studies applied the VAS task developmentally. Kutlu et al. (2024) found that children with more diverse linguistic input (i.e., hearing different languages, accents, and dialects) showed more gradient categorization, supporting flexibility, though older children were slightly more categorical. Kim, Klein‐Packard, et al. (2025) found that dyslexia and language disorder were linked to greater response variability (lower consistency) but not slope differences, suggesting consistency may be a key developmental dimension. Adult work also suggests that categorization consistency is a robust individual trait (Kim, McMurray, et al. 2025). Thus, consistency—which has not been examined developmentally—may be an equally important dimension to the slope of the function.

### 1.4 The Present Study

This study used the VAS task to precisely characterize the dimensions of speech categorization that develop from 1st through 6th grade. We tested school‐aged children in a set of VAS tasks spanning a large set of speech contrasts to address three questions. First, what is the developmental timecourse? Second, as a whole, are children becoming more gradient or categorical? And third, are there developmental changes in categorization consistency?

We extracted two indices from the VAS task to capture children's speech categorization profiles. First, we estimated each child's categorization *slope*. This is analogous to slope in traditional forced‐choice tasks. Second, we estimated trial‐by‐trial *response variability*, which captures the degree of categorization consistency. High response variability means more inconsistency in responses from trial to trial, while low response variability means a more consistent/less noisy response pattern.

If the target of development is toward a more discrete category structure, the most important changes will be in slope: children should become steeper with development (more categorical), with a potentially ancillary increase in consistency (since it is impossible to show a steep slope with high variability). Alternatively, if children are becoming more gradient, we expect primarily reductions in variability, with potentially a reduction in slope, as categories adapt to the fine‐grained structure in the environment. However, we also examined categorization consistency as a potential second locus of development.

## 2 Methods

This experiment was part of a large, multi‐year longitudinal project, *The Growing Words*. This project assessed many aspects of speech perception, word recognition, language, and reading; a subset of these measures is used here. Recruitment and experimental procedures were approved by the University of Iowa Institutional Review Board (IRB#201809789). All participants’ legal guardians provided written consent, with children providing verbal assent. Participants received $15/h during the first year, and this rose each consecutive year to enhance retention. All data and scripts can be found on our OSF repository (https://osf.io/rws9k/?view_only=4de08e7f77b94b7c85206f184c1f9a26).

### 2.1 Participants

Participants were monolingual American English‐speaking children with normal hearing and normal or corrected‐to‐normal vision and no major intellectual or developmental disabilities. Since the goal of the larger longitudinal study was to examine variation in language and reading profiles, we did not exclude children with developmental language disorder or dyslexia. This was motivated in part by the fact that these so‐called disorders are widely seen as the lower end of a spectrum of ability (Leonard 1991; Shaywitz et al. 1992; Tomblin and Zhang 1999). Other major developmental disabilities (e.g., Autism, Down Syndrome), which may also affect language, but which are linked to more pervasive cognitive differences, were excluded.

To diversify our sample, we conducted experiments at two sites, Iowa City (a small college town and the location of the University of Iowa) and Cedar Rapids (a larger and more diverse city approximately 30 mi north of Iowa City). All participants were tested in an identically configured lab in both locations.

Growing Words uses an accelerated longitudinal design in which three cohorts of children were recruited, starting the study in 1st, 2nd, or 3rd grade. We use grade as the primary factor, rather than chronological age, as a key focus of Growing Words is on reading development. With 4 years of testing, this means that adjacent cohorts overlap at three test points, and all cohorts overlap at two. Children are tested between January and August of each year, which started in 2021 and ended in 2024.

Participants were recruited through various methods, including in‐school screenings, emails to the university community, and direct marketing at community centers and farmers' markets. Several participants were dropped from the analysis because of speech‐related issues (*N* = 2), bilingual exposure (*N* = 2), attentional and behavioral issues (*N* = 1), failure to complete the second session within the same day (*N* = 3), and those who started the longitudinal study in Year 4 (*N* = 2). We also excluded some participants from the analysis due to inconsistent/flat slope fits based on the exclusion criteria (i.e., asymptotes outside the range of 25%–75%) (*N* = 16). In addition, we excluded participants who did not participate in all years of testing and whose data were not processed accurately, which led to the loss of data (*N* = 39). Consequently, the final sample size used here was 225. Demographic information about the sample—as a function of both cohort and location—is shown in Table 1.

**TABLE 1:** Distribution of participants in various demographic categories.

	Cohort			Location	
	Grade 1	Grade 2	Grade 3	Iowa city	Cedar rapids
**Race**					
Asian	1.4%	2.6%	2.6%	1.8%	2.7%
Black or African American	5.7%	3.9%	5.1%	3.6%	6.2%
White	87.1%	90.9%	89.7%	91.1%	87.6%
Other	5.7%	2.6%	2.6%	3.6%	3.5%
**Ethnicity**					
Hispanic	8.6%	2.6%	5.1%	4.5%	6.2%
Not Hispanic	91.4%	97.4%	94.9%	95.5%	93.8%
**Gender**					
Female	47.1%	49.4%	39.7%	44.6%	46%
Male	52.9%	50.6%	60.3%	55.4%	54%
**Household income**					
<$25,000	0%	1.5%	0%	0%	0.9%
$25,000–$34,999	1.5%	0%	1.3%	0%	1.9%
$35,000–$49,999	6.2%	10.3%	7.9%	3.9%	12.3%
$50,000–$74,999	13.8%	5.9%	13.2%	8.7%	13.2%
$75,000–$99,999	15.4%	17.6%	10.5%	12.6%	16%
$100,000–$149,999	33,8%	35.3%	40.8%	42.7%	31.1%
$150,000–$199,999	16.9%	14.7%	19.7%	20.4%	14.2%
$200,000–$249,999	4.6%	11.8%	1.3%	7.8%	3.8%
Prefer not to answer	7.7.%	2.9%	5.3%	3.9%	6.6%
**Maternal education**					
High school or GED completed	0%	2.6%	1.3%	0.9%	1.8%
Associate degree (AA, AS)	7.1%	9.1%	5.1%	1.8%	12.4%
Some college credit but no degree	8.6%	7.8%	5.1%	1.8%	12.4%
Bachelor's degree (BA, BS, AB)	44.3%	39%	47.4%	42%	45.1%
Graduate or professional degree	40%	41.6%	41%	50%	31.9%

*Note*: The two locations did not differ from one another statistically, except for Graduate or professional degrees (i.e., Cedar Rapids having slightly fewer mothers with graduate degrees).

### 2.2 Stimuli

#### 2.2.1 Auditory Stimuli

Auditory stimuli used in VAS experiments consisted of monosyllabic minimal pairs in English. We purposely tested a diverse range of phonemic contrasts to achieve a broader view of speech perception as a whole. Starting from that premise, we selected five minimal pairs with the following constraints: (1) both words were likely to be known by first graders; (2) both words were picturable (which was necessary for the task); and (3) existing speech synthesis methods could be used to construct phonetically realistic continua from natural speech (synthetic speech can be problematic for some children: Coady et al. 2007). Thus, the final set of continua included *beach‐peach*, *time‐dime*, *net‐nut*, *hat‐hot*, *sip‐ship*. For each minimal pair, a seven‐step continuum was created using distinct methods for each contrast type.

For all five continua, the process started by recording each endpoint spoken by the same phonetically trained male talker of American English in a sound‐attenuated room. The talker repeated each word 8–10 times in a neutral carrier phrase (“He said…”) at a relatively slow rate of speech. We selected a single exemplar for each word, balancing clarity, lack of artifacts, and overall acoustic similarity to the other word (e.g., *beach* and *peach* should have a similar length and prosody). These were then excised from the carrier sentence.

Next, we constructed seven‐step continua. For the voicing continua, seven steps were created by replacing segments of voicing at vowel onset for the voiced end of the continuum with equal‐sized segments of aspiration from the voiceless side (McMurray et al. 2008). For the vowel continua, Tandem STRAIGHT was used (Kawahara et al. 2008). This first extracts periodic information for each endpoint. Then, temporal anchors are manually placed at the beginning, middle, and end of the target sounds. Finally, continua were morphed across two endpoints in seven steps. For the *sip/*ship continuum, a spectral averaging procedure was used based on prior studies, implemented in MATLAB (Galle et al. 2019) and used in our prior developmental work (McMurray et al. 2018). First, the frication portion from *sip* and *ship* was extracted from the full words. Then, the longer of the two was cut so that they would have equal length. In the third step of the process, the /s/ and /ʃ/ spectra are shifted to have the same center frequency; these were then averaged in various increments so that the overall shape of the spectra (e.g., how peaky or wide it is) varies with each continuum step. Next, these shapes were shifted to have peak frequency aligned at the appropriate point between the original two peak frequencies for that continuum (assuming linear spacing). Finally, the resulting spectra were used to filter a segment of white noise, which had an envelope that was the average of the /s/ and /ʃ/ endpoints.

#### 2.2.2 Visual Stimuli

Pictures of each endpoint were shown at either end of the VAS to convey the nature of the scale. These visual stimuli were created using a picture‐norming process adapted from previous studies (McMurray et al. 2010). For each item, a set of 3–5 candidates was downloaded from a commercial clipart dataset. These were then discussed by focus groups of laboratory members to select the prototypical image and recommend any changes (e.g., for a more consistent style, orientation, etc.).

### 2.3 Procedure

Each year, Growing Words tests children over 2 days in the laboratory and one shorter Zoom session. Each laboratory session lasted approximately 2–2.5 h (see https://osf.io/vzb2k/ for the complete design of Growing Words). VAS testing was interspersed across both days to avoid fatigue.

On each day, VAS testing started with three practice trials. These were identical to the experimental trials and were intended to orient the child to the general task. After practice, an experimenter ensured that the child understood the task, and then the experimental trials began. On each trial, participants saw two images along with the VAS line. They then heard the auditory stimulus and tapped on the scale to indicate where the sound fell (participants did not report any issues with tapping, as all were familiar with touch screen systems). To avoid biasing their responses, the line did not contain a slider or any marker until the participant responded. Participants could change their responses by tapping on a different location on the scale. Once they were satisfied with their response, they hit the space bar to save it and initiate the next trial.

Trials were blocked by continuum to minimize the cognitive effort it would take to remap the scale to new words and/or continua on each trial. To control for order effects and image‐position bias (i.e., seeing a *beach* image on the right), there were two blocks of trials for each continuum (one on each day) and the position of the images was swapped on the second day (e.g., if *beach* was on the left on Day 1, it was on the right for Day 2). Within a block of trials, the order of the steps was randomized. Each participant completed 28 trials (seven steps, 2 days, two repetitions) for each continuum for a total of 140 (28 × 5 continua) experimental trials, requiring about 10 min split across days. Continua were presented in the following order: On Day 1, *beach/peach, net/nut, hat/hot, dime/time, ship/sip*; on Day 2: *dime/time, hat/hot, net/nut, beach/peach, ship/sip*.

### 2.4 VAS Data Processing

On half the trials, the items (pictures) were on the opposite side of the scale (e.g., if “beach” was on the right for the first block of *beach/peach* trials, it moved to the left for the second). Thus, VAS scores were transformed (by subtracting the rating from 100 for half the trials) so that high values always corresponded to step 7 of the continuum.

We next checked each participant's mean responses as a function of continuum step for each year. We specifically excluded datasets where asymptotes failed to reach 25 and 75, respectively. At levels below that, we could not be certain whether children are failing to accurately perceive the endpoints as clear category members, or whether they misunderstood the task (or both). This exclusion ensures that we only analyzed data where we could be sure that participants met the minimal criteria for valid data. However, it also means that we cannot make strong claims about the degree to which there is development in asymptotic performance (on clear exemplars) in this time window, as has been observed with younger children (Creel 2022; Lalonde and Holt 2015). Exclusion was year‐specific (e.g., a participant could be dropped for Year 2 but retained for Years 1, 3, and 4). This resulted in the exclusion of an additional 13 data points. This left a final dataset that included 841 measures (participants × years), with an average of 3.71 samples for each subject.

We modeled VAS responses with a four‐parameter logistic function mapping continuum step (1–7) to VAS score (0–100). This function, common in speech perception research, estimates slope and boundary, and free upper and lower asymptotes. It is well‐suited here because (1) children may not rate endpoints at extremes (0 or 100) due to developmental or task‐related limits, and (2) it separates slope from asymptotic performance, unlike two‐parameter models where non‐ideal asymptotes distort slope estimates.

The logistic function was embedded in a mixed‐effects framework, with slope, crossover, and asymptotes modeled as subject‐ and item‐level random intercepts. This approach fits all data jointly, improving parameter estimates—e.g., data from one continuum (*dime/time*) informs fits for another (*beach/peach*)—which is especially useful given our smaller trial counts to accommodate children's attention spans. Because of the model's nonlinearity, we estimated parameters using Bayesian rather than conventional maximum‐likelihood methods.

We fit this Bayesian non‐linear mixed effects model to the VAS data using a custom approach developed by Sorensen et al. (2024) for the VAS task and implemented in the *rstan* package (Guo et al. 2020) in R (see Sorensen et al. 2024 for details). The Bayesian model was used to fit the four‐parameter logistic as described with random intercepts by subject and item for the lower and upper asymptotes, the crossover or boundary, and the slope at the boundary. The mixed model also included a fifth parameter (as a random intercept) that captured the trial‐by‐trial variance (log scaled) around the curve. This was modeled as a quadratic function over the VAS steps to account for the fact that trial‐by‐trial variance was dependent on the continuum step (variance would necessarily be higher in the middle of a continuum).

This model was fit to each year separately (the complexity of the model precluded a longitudinal approach). Once fit, we estimated individual subjects’ random intercepts. For subject‐specific slope and response variability estimates, see Supplement S1 for details of how this was done. Finally, these indices were submitted to longitudinal growth curve analyses in a standard linear mixed effects framework, as described in the results. This was done with the *lme4* package (Bates et al. 2015) to run the statistical models and *easystats* package to extract model fits, including the standardized betas (Lüdecke et al. 2023).

In this parametrization, the slope is defined as the derivative of the function at the midpoint (e.g., the change in VAS responding for a single step of the continuum). The boundary is defined in terms of the continuum step, and the amplitude will scale from 0 to 100. The response variability is more difficult to describe on an absolute scale as it is derived from a function in the mixed model, but it roughly scales in units of 100 (the points along the VAS scale), where 0 would indicate no variability and values of 500–600 are possible.

## 3 Results

**FIGURE 1:**
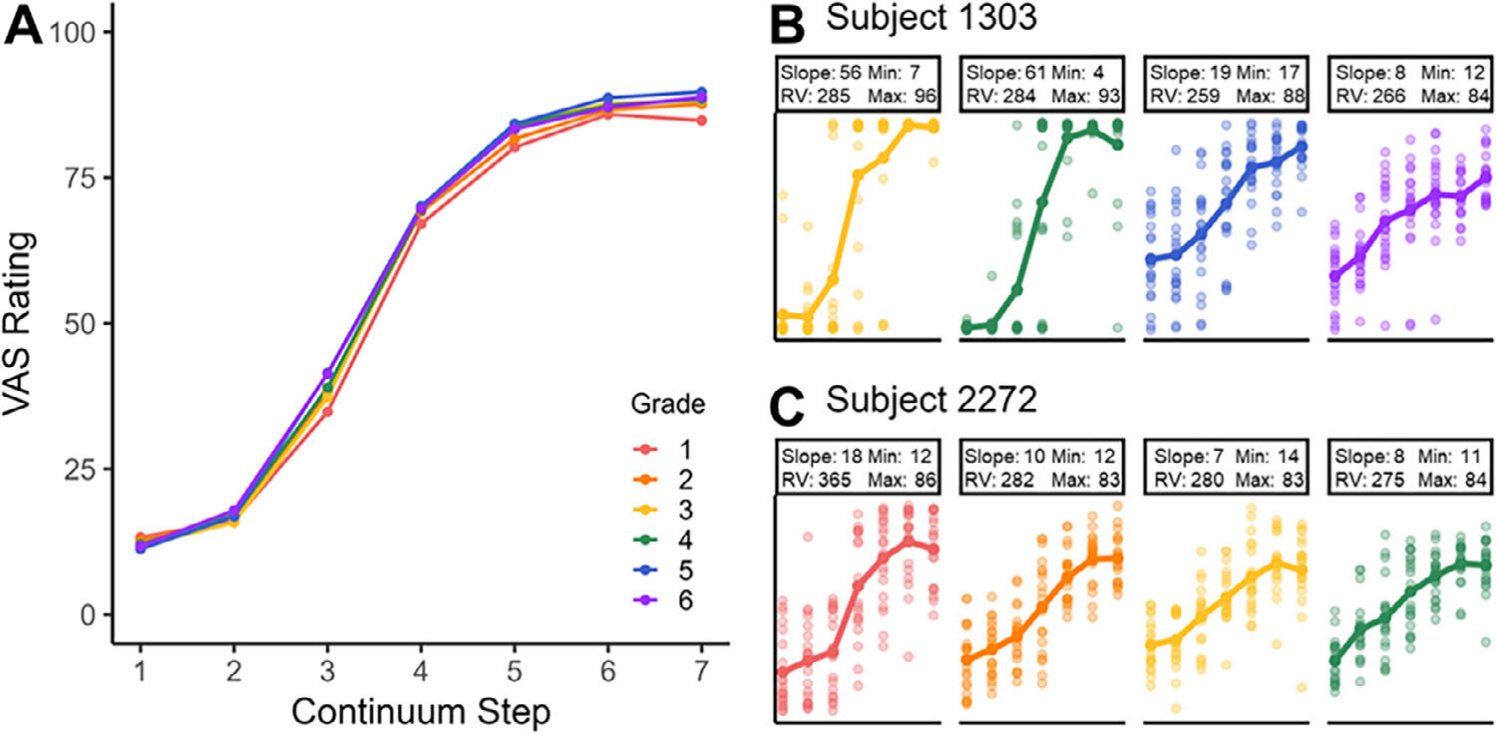
Two subjects’ data shown across different grades (see the legend in panel A for each grade). Each participant's Slope and Response Variability scores were presented along with the visualization. This visualization is averaged over continuum.

Figure 1 shows VAS responses across each grade as a function of VAS step. This suggests clear grade differences from Grade 1 to 6, such that as grade increases, the slope steepens. This aligns with previous observations from forced‐choice tasks (Hazan and Barrett 2000; McMurray et al. 2018), though it significantly expands the developmental grain with year‐by‐year changes. We also depict yearly data for two participants alongside their estimated Slope and RV values (subjects 1303 and 2272) in Figure 1B,C to help map the numerical estimates onto actual patterns of data. Each color represents their grade. These suggest that at an individual level there are likely to be changes in both the average function (slope) and the trial‐by‐trial variance.

Although the mean function shows steepening, recall that the overall mean slope of the function is a product of both the true underlying gradiency and the trial‐by‐trial variability. That is, if response variability is decreasing, the mean may look more categorical. In fact, when we separate the contributors of this pattern by estimating the slope and response variability in the mixed framework, we see a different picture.

### 3.1 Slope

A growth curve model was fit to the Slope (using a linear mixed effects framework). Fixed effects included testing year (1–4, within‐subject, centered) and participants’ start grade or cohort (1st, 2nd, 3rd, as a linear term, also centered) along with their interaction. We used the maximal random effects structure with a random intercept and slope of testing year on participants.

**FIGURE 2:**
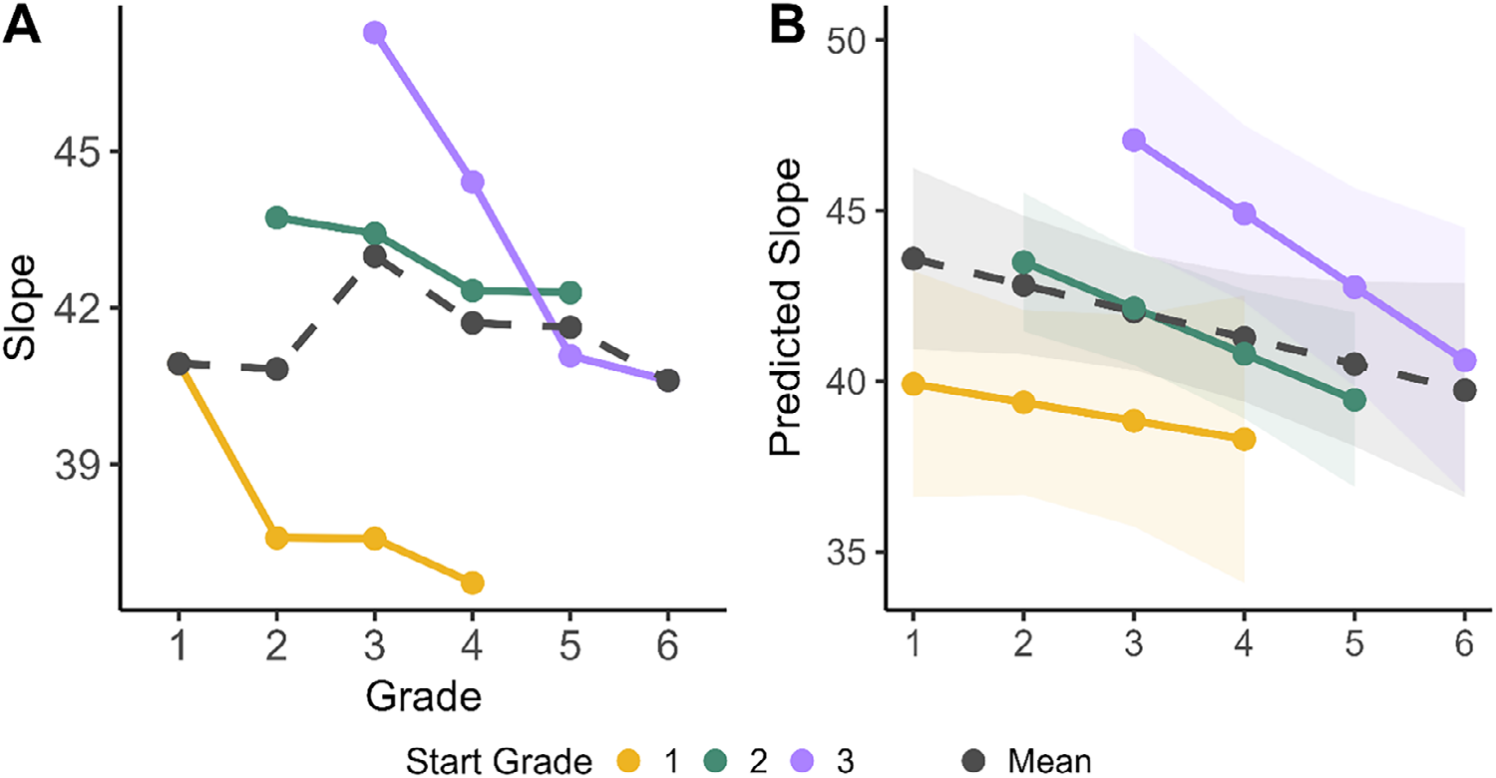
(A) Slope estimates (y‐axis) across grades along with one averaged line for all groups. (B) Predicted Slope values from the Slope model on the y‐axis as a function of years in study. Each line shows a separate start grade group. Both graphs showing a decrease (shallowing or toward more gradient categorization) of the Slope function.

The results revealed significant effects of testing year and start grade on Slope. Longitudinally, from Year 1 to 4, children's slopes became more gradient (less steep) (B = −1.39, 95% CI [−2.42, −0.36], *t*(827) = −2.64, *p* = 0.008; *β* = −0.08, 95% CI [−0.15, −0.02]). Conversely, for the start grade, children who started the project at older ages had significantly steeper slopes (B = 2.42, 95% CI [0.34, 4.51], *t*(827) = 2.29, *p* = 0.023; *β* = 0.11, 95% CI [0.02, 0.20]) (see Figure 2). To put this in perspective, a younger child (with a steeper slope) might give very similar VAS ratings to two tokens that are both clearly on the /p/ side of a continuum but differ in VOT (e.g., two tokens at 40 and 60 ms might both be rated around 90). In contrast, whereas 4 years later, the same child would rate a moderately aspirated token lower (say, 80) and a heavily aspirated token higher (95). In other words, older children differentiate sounds within the same category more than younger children do, reflecting a shallower slope.

The interaction between testing year and start grade was not statistically significant (B = −0.81, 95% CI [−2.09, 0.47], *t*(827) = −1.24, *p* = 0.217; *β* = −0.04, 95% CI [−0.10, 0.02]), implying that the relationship between testing year and Slope does not significantly differ across different starting grades. Thus, the effect of cohort was present at all study years and did not seem to moderate the pattern of growth (see Figure 2).

### 3.2 Response Variability

**FIGURE 3:**
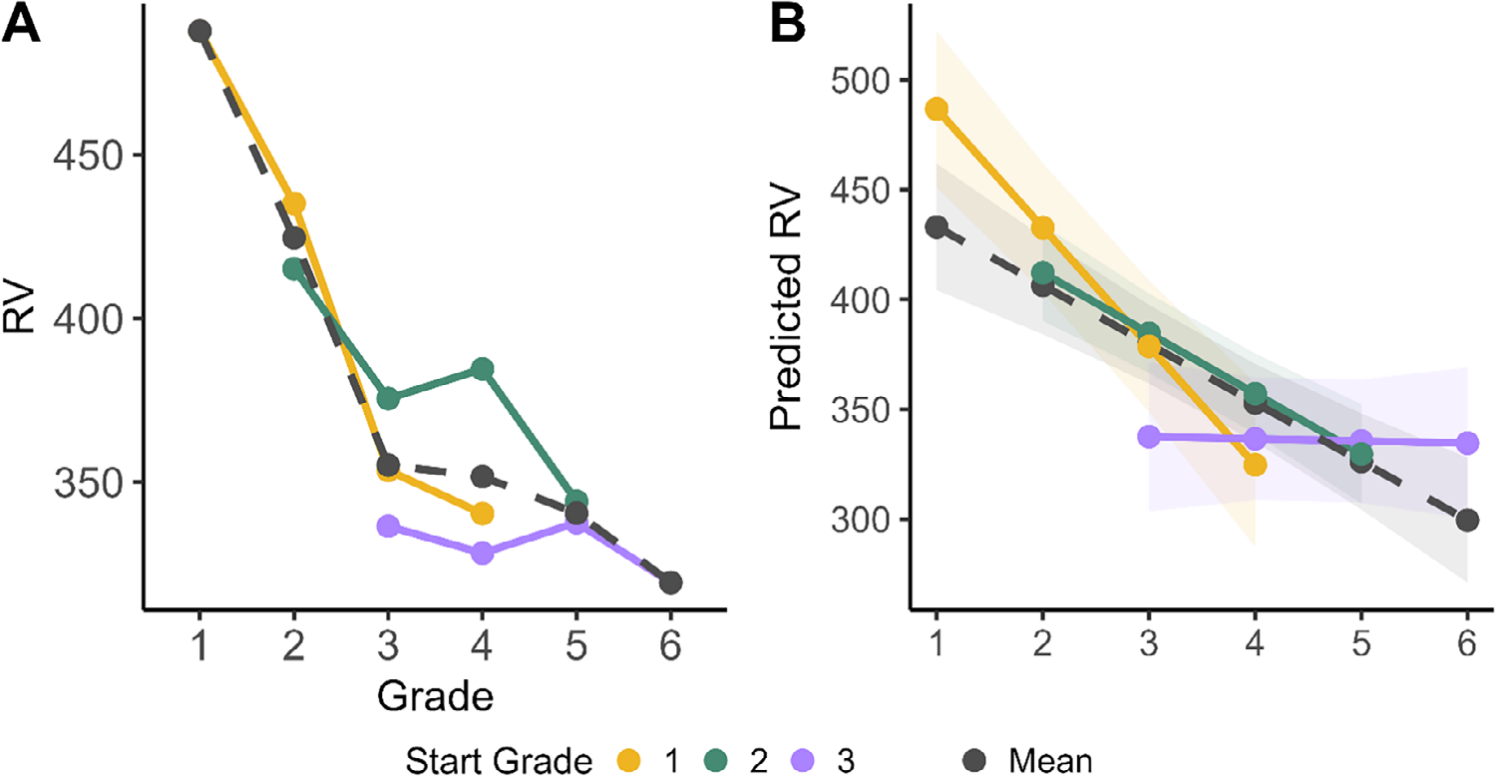
**(A)** Response variability across grades with one averaged line for all groups. On the X axis, true grade is represented (i.e., the longitudinal changes). The colored lines represent the start grade for each participant. (**B)** Predicted values from the Response Variability model on the y‐axis as a function of years in study. Both visualizations show clear decrease in Response Variability (i.e., children become more consistent).

A second growth curve model was fit to Response Variability with the same fixed effects (i.e., testing time, start grade) and random effects structure. The results revealed that both predictors had a significant negative effect on Response Variability. From testing year 1 to 4, Response Variability significantly decreased (B = −26.08, 95% CI [−35.13, −17.03], t(827) = −5.66, *p* < 0.001; *β* = −0.16, 95% CI [−0.21, −0.10]), meaning that children become more consistent in their responses. The same decrease was observed as a function of start grade (B = −36.78, 95% CI [−58.39, −15.18], t(827) = −3.34, *p* < 0.001; *β* = −0.16, 95% CI [−0.26, −0.07]). The interaction between test year and start grade was also significant (B = 26.52, 95% CI [15.28, 37.77], t(827) = 4.63, *p* < 0.001; *β* = 0.13, 95% CI [0.07, 0.19]), suggesting that the relationship between testing year and Response Variability significantly differs across cohorts. A follow‐up analysis using the ggeffects package (Lüdecke 2018) shows that for the older start grades, the Response Variability plateaued: essentially, the rate of change was tapering off as children became older (see Figure 3). This suggests a stabilization in the system around Grade 4–5 (see Figure 3).

Note that some variance in children's VAS task performance could reflect general task engagement rather than changes in speech perception. However, analyses in Supplement S4.1 show similar developmental patterns after controlling for attention, cognitive control, and performance on unambiguous items, and rule out task‐specific learning effects. Thus, observed changes in slope and response variability likely reflect true perceptual development.

## 4 Discussion

This study used a novel speech categorization task (VAS) to examine the developmental trajectory of speech categorization. We first consider whether our findings might reflect task‐specific or attentional factors rather than speech perception per se. The study addressed three main questions. First, we traced the developmental course of speech categorization and found that it continues to mature across multiple dimensions through 6th grade. Second, we asked whether development reflects a shift toward more gradient or categorical speech representations, using the slope of the categorization function as our index. Our primary evidence comes from longitudinal changes within subjects over 4 years, though some cross‐sectional results across cohorts were less consistent. Thus, we discuss within‐ and between‐subject effects on slope separately. Finally, we explored whether other dimensions of speech categorization develop during this period, identifying a new measure—categorization consistency—which captures the stability of perception from trial to trial. We conclude by discussing the broader implications of these findings for speech and language development in school‐age children.

### 4.1 Task or Attentional Effects

Before interpreting these results, it is important to consider whether developmental differences could reflect general task engagement or limitations in children's ability to perform the VAS task, rather than changes in speech perception itself. Although we cannot entirely rule out such effects, converging evidence from previous research and additional analyses (see Supplement S4.2) suggests these factors do not fully account for our findings.

One concern is whether children understood the task. Ideally, an accompanying VAS task using a simple, continuously scaled factor would confirm this, but it was not feasible in this study. However, prior research indicates that children as young as 6–7 can understand and use VAS scales appropriately (Shields et al. 2005, 2003).

Work from our research group further supports this: Found that adult categorization consistency in speech perception predicted 30% of the variance in language skills, even after controlling for variability on a non‐linguistic visual continuum. Similarly, Kim, Klein‐Packard, et al. (2025) reported that, in third graders, response variability was linked to language and reading skills, but not to cognitive or self‐regulation measures. (Kim, Klein‐Packard, et al., 2025). Moreover, this relationship was not mediated by meta‐phonological awareness—such differences do not reflect general ability with sound nor the ability to do cognitively difficult tasks (as phonological awareness tasks are difficult for many children). These findings point to a speech‐specific, rather than domain‐general, basis for our effects.

Additional analyses (see Supplements 2–4) further address potential confounds. First, adding parent‐reported self‐regulation and cognitive control to growth curve models (Supplement S4.1) did not reduce the developmental effects on response variability. Core effects of years in study, start grade, and their interaction remained stable in both magnitude and significance. Second, to assess whether attention or knowledge of endpoint stimuli (unambiguous tokens) influenced results, we examined asymptote responses (Supplement S2), finding no developmental differences. Repeating analyses using only participants who rated each endpoint as clearly unambiguous (Supplement S3) again produced robust developmental effects.

Another possibility is that children simply became more gradient in their responses due to practice with the VAS task itself. If the improvements were driven by task familiarity, we would expect the largest change to occur immediately between the first and second session once children learned how to use the scale. Conversely, if the change reflects real perceptual development, we would expect gradual improvements over the years. We examined this by comparing the first session to later ones, and found only a steady, continuous change rather than a one‐time jump (see Supplement S4.2). This pattern is consistent with ongoing developmental change rather than an acute task‐specific learning effect.

Although we cannot entirely dismiss all task‐related concerns (e.g., younger children's less mature motor control added noise to their VAS responses), several factors suggest motor issues are not the primary driver of our effects. Children could adjust the slider until satisfied (minimizing simple motor errors), and prior work shows even 6‐year‐olds can use such scales effectively (Shields et al. 2005, 2003). Moreover, the consistency gains continued through the oldest ages tested, indicating improvements beyond basic motor skill acquisition. We have nevertheless noted that future studies should consider motor demands (e.g., future work should include parallel non‐linguistic VAS tasks or test VAS use with visual continua, or consider tasks that assess pure motor variability).

### 4.2 Toward a More Gradient System

Our second research question asked whether children develop increasingly gradient or categorical representations of speech categories. This was motivated by findings that younger children and those with language or reading impairments show shallower slopes in FC + C tasks, in contrast to the adult literature, where a more gradient (shallower slope) system is seen as typical and potentially advantageous. We found strong longitudinal evidence for development toward a more gradient system, despite some puzzling between‐subject effects.

#### 4.2.1 Within‐Subject Development: Increasing Gradiency in Speech Categorization

This study is the first longitudinal investigation of speech categorization in school‐aged children, revealing significant within‐subject decreases in slope. This indicates that, over time, children do not become more categorical; instead, they increasingly attend to fine‐grained acoustic details. These findings challenge models that emphasize growing categoricalness with age, and instead support a developmental trajectory where children progressively adopt a more gradient approach to phoneme categorization.

This trend is supported by prior work showing that even infants exhibit gradiency (Galle and McMurray 2014; Miller and Eimas 1996), suggesting that the narrowing often observed in infancy may be more about the task than a true developmental shift. Traditional perceptual narrowing theories, originally meant to describe native versus non‐native category attunement, predict that children start with a degree of perceptual openness (which is analogous to our gradiency) and become more restricted (∼categorical) over time as they learn what details matter. However, a more nuanced view (Werker 2018) proposes a U‐shaped trajectory: infants start out with a gradient, narrow in sensitivity during infancy, and then become more gradient again later in childhood.

Several points challenge the U‐shaped hypothesis. First, two studies of infant VOT sensitivity (McMurray et al. 2008; Miller and Eimas 1996), and meta‐analytic evidence (Galle and McMurray 2014), show infants demonstrate gradiency (and may even increase it) through about 18 months—similar to the trends observed here. Second, methodological issues with FC + C tasks complicate interpretation, as slope may not accurately reflect underlying representation. More sensitive tasks (like VAS or VWP) might reveal different patterns, exposing ambiguity in how task data maps to underlying cognitive structures (see McMurray 2023).

A more parsimonious account is that children, from infancy through adolescence, become increasingly sensitive to fine‐grained details. What appears as “narrowing” in infancy may instead reflect interpretive narrowing—paying more attention to the categories of their language (as opposed to other non‐categorical distinctions), even as the ability to make these contrasts is not lost (Bergmann et al. 2018; Hay et al. 2015). This view aligns with our findings and with the broader attunement framework (Werker 2024), which suggests category acquisition involves learning graded, prototype‐like structures rather than strict boundaries. If distributional learning underlies attunement, then the persistence of gradient learning for years is exactly what would be expected, given the highly gradient nature of speech cue distributions.

Further progress will require: (1) tasks that directly assess gradiency in infants, especially with reliable single‐trial measures; and (2) developmental continuity across the critical 1–5‐year‐old window, where current tasks and measures often differ.

More broadly, developmental models emphasizing increasing categoricalness have long conflicted with the adult psycholinguistics consensus that speech perception is gradient and that gradiency is functionally beneficial (McMurray 2022). Our evidence for increasing gradiency with age helps reconcile these views and suggests that the functional benefits of gradient perception for flexible listening are a developmental achievement.

#### 4.2.2 Between‐Subject Effects: The Influence of Language Environment

Although our main focus was on within‐subject developmental changes, we also observed between‐subject effects that contrasted with the pattern of increasing gradiency: across cohorts, older children showed steeper slopes. Notably, such between‐subject effects have been reported elsewhere; Kutlu et al. (2024) found similar steepening of VAS slopes between ages 6 and 11 in a comparable timeframe.

One possible explanation is the influence of the language environment. Laboratory learning studies indicate that the gradiency of speech categories adapts to the statistical variability of input: more variable input leads to more gradient categories (Clayards et al. 2008; Theodore and Monto 2019). These experiments manipulated the VOTs on each trial such that the VOTs were randomly chosen from a bimodal Gaussian distribution (one peak for each consonant category), using either low variance (categories that barely overlap) or high variance (categories that highly overlapped). Listeners in the high variance condition tend to show more gradient effects. Critically, a recent laboratory learning study (Gür and McMurray 2024) suggests that VAS gradiency—but not response variability—is what shifts in these abruptly changing conditions, paralleling our cohort effects on slope but not response variability.

These raise the possibility that such learning effects could play out in the real world. Indeed, Kutlu and colleagues (2024) further showed that children exposed to more diverse linguistic environments (e.g., a variety of dialects or accents) exhibit shallower VAS slopes, reflecting more gradient perception; those in less diverse environments respond more categorically. So how could such effects account for our cohort differences?

Our longitudinal study began shortly after the onset of the COVID‐19 pandemic and the social isolation that many families experienced as a pandemic‐related caution. Our study thus tested children who encountered dramatic changes in the language environment right around the time of testing. Consequently, these sorts of in‐lab experiments (e.g., Gür and McMurray 2024) may capture something akin to what our participants went through when they started school and had to abruptly change their schooling environment (i.e., change in the distributions) back to the home environment.

In our case, third graders had at least 2 years of diverse linguistic input (at school) before the pandemic, followed by a more isolated experience just before we began testing. In contrast, the first graders would have had more limited exposure in home or small preschool settings prior to the pandemic, followed by isolation during it. Thus, the older children may have experienced greater disruption—a sudden loss of diversity—prompting a shift toward more CP. In contrast. the younger cohorts might not have shown such effects, as their experience was more consistent. Older children in our study had to rapidly adjust from home to school and from school to home setting. And this might have been exacerbated by the possibility that older children adapt more rapidly to current conditions than younger children (e.g., (Fuhrmeister et al. 2020))

Although we do not provide concrete data on the amount of input these children receive during COVID‐19 (e.g., our participants came from a range of school districts, including private and home schools, and we lacked individualized data on their schooling during the pandemic), it is likely that online schooling reduced phonetic diversity and overall speech input. This was followed by a dramatic increase as schools reopened—timing that aligns with both our study and the one previous study showing between‐subject increases in slope for a new group of children tested right around the same time (Kutlu et al. 2024). Thus, the older children may have experienced greater disruption—a sudden loss of diversity—prompting a shift toward more CP.

Although our interpretation of the direction of effects is necessarily post‐hoc, it is notable that only slope—not response variability—showed these cohort effects. Although we cannot fully explain these between‐group differences, they highlight the substantial individual differences in speech categorization arising from environmental factors, underscoring the value of longitudinal research. Importantly, despite cohort differences in slope, all groups showed the same within‐subject effect: regardless of their starting point, over the 4 years of the study, children's slopes became more gradient.

### 4.3 Categorization Consistency: A New Dimension of Speech Categorization

A key—and somewhat surprising—finding was the dramatic, consistent change in categorization consistency. Unlike slope, response variability showed a clear developmental pattern: both between‐ and within‐subject reductions as children aged. This suggests that as children mature, they become more stable in their speech categorization, reliably interpreting the same stimulus across trials. Notably, this dimension of speech categorization cannot be measured with traditional forced‐choice tasks and has been largely overlooked in prior developmental research.

Although these differences might reflect domain‐general abilities, they have been linked to language and reading outcomes, not cognitive regulation (see Supplement S4.1). Thus, while we cannot definitively rule out domain‐general explanations, our results support the view that these changes reflect a fundamental aspect of speech perception.

Response variability—as a measure of categorization consistency—appears more psychometrically robust than slope, correlating across different continua and predicting a range of outcomes (Kim, Klein‐Packard, et al., 2025). Across years and grades, we observed a significant decrease in response variability, indicating that children became more consistent in their categorization. This developmental change is robust, in contrast to the more complex pattern observed for slope, and highlights that, as children age, their interpretations of the same stimulus become more stable. This pattern is not predicted by accounts focusing solely on category structure; instead, it points to dramatic improvements in the consistency of the perceptual processes underlying phoneme categorization.

Categorization consistency is not well addressed by existing models of speech perception, which generally emphasize phonemic representations and the mechanisms by which they are learned (Feldman et al. 2013; Nixon 2020; Schatz et al. 2021). These models do not account for what may be a crucial dimension of both development and individual differences. Dynamic systems and hybrid approaches (Case et al. 1995; McMurray, Horst, et al. 2009) may offer a framework for considering perceptual stability, though this has yet to be formally developed.

What drives this development in consistency? Insights come from auditory neuroscience: the consistency of brainstem responses to speech shows developmental change (Skoe et al. 2015). Our findings may tap into similar mechanisms, though brainstem response typically stabilizes after age 8, while our results show continued improvement, suggesting additional cortical involvement.

Several mechanisms may underlie these developmental changes. First, they may be input‐driven: exposure to phonetically diverse input predicts both more gradient categorization (shallower slope) and reduced variability (Kutlu et al. 2024). This suggests children may tune their consistency in response to environmental demands. This slow learning may be especially relevant for older children as their social networks and linguistic demands grow.

Developmental pressures may also play a role. As children's vocabularies expand and they encounter more diverse speakers and more complex language, demands on speech perception increase. This may drive the system to become more consistent, though the precise mechanisms remain unclear (Walley et al. 2003).

Importantly, top‐down processes may contribute to increased consistency. Children might use lexical knowledge, phonological rules, orthography, or familiarity with talkers to stabilize their perceptions (e.g., Magnuson et al. 2024). Gains in these higher‐level linguistic skills may be critical for developing consistency in speech perception.

### 4.4 Speech Development in Childhood

Our findings show that the development speech categorization is not a matter of sharpening phoneme boundaries. Rather children appear to be increasing gradient sensitivity to phoneme contrasts. Moreover, this is accompanied by even larger changes in a new dimension: consistency.

These two dimensions emphasize changes near the middle of the continuum—arguably the place where children need the most sensitivity to fine‐grained differences in order to accurately delineate categories and account for context. However, children may also develop at other locations along the continuum that we cannot detect.

First, our study was not designed to examine changes in the representation at the category prototypes (e.g., the endpoints). This would have been expected to affect the asymptotes of the function; Supplement S2 shows that there were no developmental effects on this measure. However, it is important to note that our sample excluded children who did not show any reliable phoneme categorization, including 16 children with near‐flat psychometric functions across all years (64 data points) and an additional 13 data points within the final sample. These datapoints were excluded because we could not determine whether they indicate that the child (at that year) could not consistently distinguish even the endpoint stimuli or simply failed to understand the task. As a result, our conclusions should be interpreted as conditional: for children who had at least minimal categorization ability (and task understanding), we see increasing gradiency and reduced noise. This may underestimate the true onset of these perceptual changes.

Indeed, there is some evidence that children's ability to recognize clear (endpoint or prototypical) tokens of minimal pairs develops. Holt and Lalonde (2012), for example, showed development between 2 and 3 years of age, but 3‐year‐olds were only at 90% correct, suggesting some room for further development. Similarly, Creel (2022) found performance on a variety of close minimal pairs to be above chance—but not yet at ceiling—by 4.5 years of age. Moreover, when we look with more sensitive measures, such as the visual world paradigm, we see that even when children's accuracy at identifying the endpoints is at ceiling, the efficiency by which they make those changes continues through at least ages 10–12 and possibly through early adulthood (McMurray et al. 2018). Thus, while our conservative approach has not been able to detect changes at the endpoints, there is reason to believe that they show a similarly protracted development.

Second, a wealth of studies by Miller (c.f. Miller 1997) for a review, have used goodness rating tasks (similar to the VAS) to examine extreme points along the continuum (e.g., /p/’s with VOTs well beyond the prototype. These reveal a graded prototype structure as well. This has not been examined developmentally, and our continuum was not sufficiently extended to do so here. However, given that this goodness structure likely derives from the statistical structure of the input, it seems likely that this too would undergo a fairly protracted development.

Putting our work in the context of this other work, it appears that sound categorization continues to develop well past the first year of life, supporting recent views of perceptual attunement (Werker 2024) and developmental continuity in language (Creel 2025; McMurray 2023). This is a significant shift for the field. For years, the first year of life was seen as the critical period for speech categorization, shaping theories of critical periods in language acquisition. However, the slow, ongoing development of speech perception—and clear evidence for plasticity into adulthood (Chiu et al. 2025; Fuhrmeister et al. 2020; Kraljic and Samuel 2006; Maye et al. 2008; Norris et al. 2003)—suggests a need to rethink these ideas (see McMurray 2023; Thiessen et al. 2016).

It is important to note that despite this continuity, fundamental speech categorization abilities are present by toddlerhood. Even 2‐ and 3‐year‐olds can reliably discriminate clear exemplars of native consonant contrasts (Holt and Lalonde 2012) and preschoolers show consistent identification of endpoint tokens of speech continua (Creel 2022; Lalonde and Holt 2015). This early stability at phoneme endpoints suggests that the basic category structure is in place by preschool age.

What develops later — as our results show through early school age — are more refined aspects of categorization: sensitivity to within‐category gradations (reflected in slope) and the stability of responses (trial‐by‐trial consistency). Our findings thus build on the toddler and preschool literature by revealing that significant changes in how children use those categories (gradiency and consistency) continue well into middle childhood.

Importantly, learning to read an alphabetic language depends on well‐formed speech sound categories. If these categories were fixed in infancy, they would serve simply as a foundation for reading. But our results indicate that speech categorization continues to develop during the years when children are learning to read and spell—suggesting these processes may co‐develop, and that literacy itself may drive further changes in speech perception (Dich and Cohn 2013). Understanding these relationships—and their implications for education and language development—remains an important direction for future research.

## Funding

The authors have nothing to report. This work was supported by National Institutes of Health Grant DC R01 DC008089 to B. McMurray.

## Conflicts of Interest

The authors declare no conflicts of interest.

## Supporting information




**Supporting File 1**: desc70085‐sup‐0001‐SuppMat.docx

## Data Availability

The data that support the findings of this study are openly available in Longitudinal Changes in the Structure of Speech Categorization at https://doi.org/10.31234/osf.io/vgybc_v1.
