# Supplementary material for: Longitudinal Changes in the Structure of Speech Categorization Across School Age Years: Children Become More Gradient and More Consistent
Source: Dev Sci. 2025 Nov 3;29(1):e70085. doi: 10.1111/desc.70085 (PMC12583890; doi:10.1111/desc.70085)
Supplement: Supplementary file 1 — Supporting File 1: desc70085‐sup‐0001‐SuppMat.docx [file DESC-29-e70085-s001.docx]

**Longitudinal Changes in the Structure of Speech Categorization Across School Age Years: Children become more gradient and more consistent.**

**ONLINE SUPPLEMENT**

| **Ethan Kutlu**  Department of Communication Sciences & Disorders  Department of Psychological & Brain Sciences  Department of Linguistics  University of Iowa  [ethan-kutlu@uiowa.edu](mailto:ethan-kutlu@uiowa.edu) |  | **Hyoju Kim**  Department of Psychological & Brain Sciences  University of Iowa  hyoju-kim@uiowa.edu |
| --- | --- | --- |
| **Bob McMurray** Department of Psychological & Brain Sciences  Department of Communication Sciences & Disorders  Department of Otolaryngology – Head and Neck Surgery  Department of Linguistics  University of Iowa [bob-mcmurray@uiowa.edu](mailto:bob-mcmurray@uiowa.edu) | | |

**S1. Parameters and Random Intercepts in the Model**

Ultimately, the goal of the non-linear mixed model was to obtain a single estimate of slope, and response variability (along with minimum and maximum values that can be calculated as the amplitude of the response) for each participant for each testing year. However, to do this correctly required the model to capture multiple sources of variance. For example, participants could have different boundaries (crossovers) or slopes for each continuum (e.g., their crossover value for the *beach-peach* continuum will be different than that of the *net-nut* continuum). It was particularly important to capture continuum-specific variance. Consider a participant who was quite categorical for each individual continuum, but who had different boundaries for each. In this case, the average function (ignoring the continuum) would have a shallow slope, even though each individual continuum was categorical. In contrast, a participant with (by coincidence) the same boundary for each continuum will look more categorical. By embedding subject and continuum level predictors of the slope and boundary in the model, this can be modeled correctly to identify the true slope, boundary (etc.) for each participant for each continuum. Thus, each of the 4 parameters in the logistic curve was modeled as a sum of four effects:

1. The population-average parameter value. This is the overall value for slope, crossover, etc., analogous to a fixed intercept.7
2. The continuum-level deviation from that. This assesses whether a particular continuum that has a higher or lower slope than average across subjects. This is analogous to a random effect of item.
3. The subject-level deviation from the average. This captures whether a given subject was higher or lower than the population value, and this is analogous to a random effect of subject.
4. The subject × continuum-level deviation. This captures whether a given continuum (e.g., *dime/time*) differed from the average for that subject.

In addition, for the log-variance, subject, continuum, and subject × continuum effects were implemented as additive effects (i.e., analogous to random intercepts) to the general log-variance function over the VAS steps.

Given this structure, to create subject-specific estimates for the slope or asymptotes, we add together the population average estimate to the individual-level deviation effects that represent the average curve for each individual across all the continua.

To obtain a single subject-specific variance estimate, the log-variance for each individual was calculated at each VAS step and averaged, quantifying an individual-specific slope and an individual-specific variance for each subject.

**S2. Developmental Effects on the Amplitude**

***Background and Rationale.*** The endpoints of the continua potentially serve as useful controls for a number of reasons. If children did not know the words or found the stimulus to be unclear, for example, they may rate even an endpoint stimulus as less good (e.g., giving it a 25 instead of a 0). Similarly, on trials where the child’s attention lapsed, they may simply guess, depressing the performance on all trials (moving ratings close to the middle) and creating more variability.

The asymptotes of the function capture the general ratings for stimuli that are not ambiguous. The minimum and maximum parameters play parallel roles in this regard: the minimum should be low and reflect the word on the “left” side of the continuum, while the maximum should be high, reflecting the word on the “right” side. None of these potential issues makes differential predictions for one word or the other, so we collapse both into a single index – the *amplitude* which reflects the difference between them. A child with robust responses to each endpoint will have an amplitude near 100, a child who is guessing or does not know the words will have a lower amplitude near 0.

We can thus assess the degree to which these kinds of factors played a role developmentally by asking whether the amplitude of the psychometric function changed developmentally. This was done by fitting a linear mixed-effects model predicting Amplitude from Years in Study, Start Grade, and their interaction, with random intercepts and slopes for Years in Study by participant.

***Results.*** The model revealed no significant main effect of Years in Study (*B* = −0.68, *SE* = 0.52, *p* = .193), suggesting that amplitude did not systematically change over the course of participation. Similarly, Start Grade did not significantly predict amplitude (*B* = 0.91, *SE* = 0.61, *p* = .138), and the Years × Start Grade interaction was also nonsignificant (*B* = 0.05, *SE* = 0.23, *p* = .825).

***Discussion.*** With the above approach, we asked whether developmental changes in speech categorization might be influenced by children’s overall ability to produce robust endpoint ratings, as reflected in the amplitude of the psychometric function. If maturation or task familiarity affected the clarity with which children identified the continuum endpoints, we would expect amplitude to increase over time or differ systematically by the age at which children entered the study.

The results did not support this prediction. Amplitude showed no significant change with Years in Study, no significant effect of Start Grade, and no interaction between these factors. Children generally produced high endpoint discrimination from the outset, and this level of performance remained stable throughout participation.

Importantly, these results do not conclusively show that children’s ability to respond to the endpoint / prototype tokens of the continuum is not developing. This is because we explicitly excluded participants (N=16) if the endpoint responding was not robust. This exclusion was necessary for both the statistical model we used, and for our methodological goal of ensuring that we are only analyzing children who clearly could recognize the words and understood the task. However, it likely artificially restricted the range of amplitudes making it difficult to detect any developmental effects.

These findings indicate that the developmental effects observed in other measures—such as a decrease in response variability—are unlikely to be driven by changes in endpoint identification ability.

**S3. Asymptote-Matched Analysis**

***Background and Rationale.*** While the prior analysis showed no overall differences in the amplitude over development, we also conducted a fine-grained approach by reanalyzing the effects on response variability but considering only children who showed robust responses to the endpoints (<10 and >90). This essentially eliminates any children who may have had these issues (e.g., they did not know the words or had lapses of attention), to ask if differences in response variability persist. Our primary analysis did essentially this already, but with a less conservative threshold (25/75).

This more conservative approach led to a sample of 178 children (the original sample included 225). These were analyzed with the same growth curves described in the main text.

***Results.*** Similar to our main analysis, the model revealed a significant negative main effect of Years in Study (*B* = −24.58, *SE* = 4.56, *p* < .001), indicating that response variability decreased with time in the study. There was also a significant negative main effect of Start Grade (*B* = −24.82, *SE* = 8.14, *p* = .002), such that children who began the study in higher grades exhibited lower variability overall. Importantly, the Years × Start Grade interaction was positive and significant (*B* = 13.36, *SE* = 5.82, *p* = .024), suggesting that the rate of change in variability over time depended on when children entered the study. Specifically, those who started earlier showed larger reductions in variability, whereas those who started later showed smaller changes.

***Discussion.*** Here, we tested whether the developmental changes in response variability observed in the main analysis persisted when considering only children with near-ceiling discrimination of the continuum endpoints. By restricting the sample to participants who consistently rated one endpoint below 10 and the other above 90, we excluded children whose performance might have been limited by various reasons (e.g., stimulus ambiguity, lapses in engagement). If the developmental effects in response variability were driven primarily by such factors, they should be greatly reduced—or disappear entirely—in this more conservative subsample.

The results did not support this interpretation. Even within this high-performing group, both Years in Study and Start Grade remained significant predictors of response variability. Children who entered the study earlier showed greater reductions in variability over time, while those starting later displayed smaller changes. The persistence of these effects in a sample restricted to children with robust endpoint performance indicates that the observed developmental improvements in response consistency are not an artifact of including lower-performing participants. Instead, they appear to reflect genuine changes in the stability of speech categorization responses over time.

**S4. Attentional and Task Related Effects**

There are a number of related concerns with any complex task used in a developmental work framework. One of these concerns is the degree to which individual or developmental differences in performance reflect the construct of interest (speech categorization) or developmental differences in simply doing the task. While there is evidence from prior studies that this task is suitable for this age group (Kim et al., under review.; Kutlu et al., 2024; Shields et al., 2003, 2005), it was possible that our results – particularly for response variability – are sensitive to children’s differential ability to stay on task and focus on a fairly complex task. Given the complexity of the task, it was also possible that learning effects are driving some of the results. Here we present follow-up analyses that offer evidence against both factors.

**S4.1 Executive Function**

***Background and Rationale.*** One concern is that differences in response variability (in particular) may reflect differences in the degree to which children can maintain attention on the task. To minimize this, the task was very short and split among two days (about 10 minutes total). Nonetheless, it was possible that differences in attention could lead to lapses on some trials.

To assess this, we used parent-reported measures of self-regulation and cognitive control from the *Behavior Rating Inventory of Executive Function* (BRIEF) (Gioia et al., 2015), and Attention Deficit subscale from the *Child Behavior Checklist* (CBCL) (Achenbach, 1999). These were entered into growth curve models predicting response variability and slope from years in study and start grade.

***Results.***

***Results: Response Variability (RV).*** When parent-reported measures of executive function and attention were added to the growth-curve model, the developmental effects on response variability (RV) remained robust. Years in Study significantly predicted lower RV (*B* = −24.82, *SE* = 4.56, *p* < .001), and Start Grade was also a significant negative predictor (*B* = −40.48, *SE* = 10.93, *p* < .001). The Years × Start Grade interaction was positive and significant (*B* = 25.84, *SE* = 5.60, *p* < .001), indicating that children who entered the study earlier showed larger decreases in variability over time compared to those who started later.

None of the parent-reported measures of executive function or attention significantly predicted RV after accounting for the developmental variables: BRIEF Behavioral Regulation (*B* = 0.06, *p* = .94), BRIEF Cognitive Regulation (*B* = 1.37, *p* = .11), and CBCL Attention Problems (*B* = 0.85, *p* = .46).

***Results: Slope.*** When predicting slope, both developmental predictors were significant. Years in Study was associated with a small but reliable decrease in slope over time (*B* = −1.47, *SE* = 0.52, *p* = .005), whereas Start Grade was positively associated with slope (*B* = 2.78, *SE* = 1.07, *p* = .010), indicating that children who entered the study at later grades tended to have steeper categorization functions overall. The Years × Start Grade interaction was not significant (*B* = −0.74, *SE* = 0.64, *p* = .248).

None of the parent-reported executive function or attention measures significantly predicted slope: BRIEF Behavioral Regulation (*B* = −0.009, *p* = .91), BRIEF Cognitive Regulation (*B* = −0.04, *p* = .64), and CBCL Attention Problems (*B* = 0.016, *p* = .88).

***Discussion.*** These findings addressed the possibility that developmental changes in speech categorization might be attributable to differences in general attentional or self-regulatory capacity, rather than growth in the underlying perceptual mechanism. If children with stronger executive function were simply better able to sustain attention and respond consistently, we would expect parent-reported measures of executive function and attention to account for a meaningful portion of the variance in response variability and slope, potentially attenuating the developmental effects.

However, this was not the case. For response variability, both Years in Study and Start Grade remained significant predictors even after controlling for BRIEF and CBCL measures. Children who entered the study earlier showed larger reductions in variability over time, and this pattern was not explained by individual differences in behavioral regulation, cognitive regulation, or attention problems. For slope, a similar conclusion emerged: developmental predictors retained their significance, whereas none of the executive function or attention measures approached significance. Older children tended to have steeper categorization functions, and slopes declined modestly with each additional year in the study, consistent with increasingly graded responses.

Together, these results indicate that improvements in speech categorization—reflected by greater response consistency (lower response variability) and more graded identification functions (shallower slope)—are not simply byproducts of better attentional control or self-regulation. Instead, the observed developmental trajectories appear to reflect genuine maturation in the targeted perceptual process, rather than the influence of broad, non-specific cognitive factors.

**S4.2 Learning**

***Background and Rationale.*** Our Visual Analogue Scaling task allows participants to continuously respond to a given stimulus. One possible concern is that children’s increasing gradiency and consistency over time might be a task-related practice effect rather than a true developmental change. For instance, children might learn to respond continuously, and they become consistent in their responses over time due to learning how to perform the task. In other words, repeated exposure to the rating task might have taught children to respond in a more gradient manner over sessions, independent of any maturation in perceptual acuity.

If this were the case, we would expect a noticeable jump in performance after the first testing session (as children become familiar with the task), followed by little change in subsequent sessions. This is motivated by classic work on skill learning where the largest gains are seen early (the power-law of practice), an effect that manifests in diverse domains of learning (e.g., Anderson, 1982; Logan, 1988), In our accelerated longitudinal design, any such practice effect at the second session would manifest as a discontinuity: a marked change in response patterns between the first and second year of testing, with minimal changes thereafter.

To test this, we fitted a linear mixed-effects growth model that partitioned time-related improvements into two components:

- **One-time practice effect at Session 2:** We included a dummy variable (i.e., followup) set to 0 at the initial session (Year 1) and 1 for all subsequent sessions (Years 2, 3, 4). This contrast captures a one-time change at the first follow-up that remains constant thereafter.
- **Continuous developmental improvement:** We included a continuous predictor for years in study (time since the first session), centered so that zero corresponds to the second session. This term models a steady linear trend over the longitudinal study period.

Each model included random intercepts for participants and random slopes for the continuous years-in-study term. We ran models for two of our key measures: Response Variability (RV) and slope of the psychometric function.

***Results: Response Variability (RV).*** The mixed model on RV revealed no significant jump at the second session. The Follow up effect was not significant (*B* = –7.4, *SE* = 16.05, *p* = .64), indicating that there was no reliable change in RV immediately upon a child’s visit after the first one.. In contrast, the continuous Years in Study effect was large and highly significant (*B* = –23.54 RV points per year, *SE* = 7.02, *p* < .001). It was also in the expected direction. As children grow up, their responses become less varied and more consistent.

***Results: Slope.*** We observed a similar pattern for the slope of the identification function. There was no significant practice effect at Session 2 (Follow up *B* = –1.40, *SE* = 1.71, *p* = .41), and no linear change over time (years in study *B* = -0.93, *SE* = 0.76, *p* = .21). The negative slope estimate implies that children’s identification functions became progressively shallower (less steep) with each additional year but this was not reaching statistical significance.

For both measures, adding the follow-up after the initial testing (i.e., practice)factor did not reveal any abrupt change specific to the second session. Instead, improvements in consistency (lower RV) unfolded as a steady, incremental trend across the multiple years of observation.

***Discussion.*** These results provide clear evidence against a task-induced practice effect. If children were simply learning the task (or learning how to respond in this task) and thus improving mainly due to familiarity, we would expect a significant drop in RV and slope at the follow-up session (reflected by a strong Follow up effect). However, no such discontinuity was found. Response variability changed at a continuous rate over time, with no jump at the second session and there was no effect of practice on Slope. This pattern suggests that the observed changes are driven by developmental progress rather than a one-time learning artifact of the task. In summary, children’s perceptual judgments became more consistent gradually as they grew older, supporting the interpretation that maturation (and accumulating experience with speech) — not just practice with the test modality — underlies the improvements in performance.

**References**

Achenbach, T. M. (1999). *The Child Behavior Checklist and related instruments.*

Anderson, J. R. (1982). Acquisition of cognitive skill. *Psychological Review*, *89*(4), 369.

Gioia, G. A., Isquith, P. K., Guy, S. C., & Kenworthy, L. (2015). Behavior Rating Inventory of Executive Function® Second Edition. *PsycTESTS Dataset*.

Kim, H., Klein-Packard, J., Sorensen, E., Oleson, J., Tomblin, J. B., & McMurray, B. (n.d.). Inconsistent Speech Categorization in School-Age Children with Language and Reading Disabilities. *Under Review*. https://doi.org/10.31234/osf.io/un6bx

Kutlu, E., Baxelbaum, K., Sorensen, E., Oleson, J., & McMurray, B. (2024). Linguistic diversity shapes flexible speech perception in school age children. *Scientific Reports*, *14*(1), 28825.

Logan, G. D. (1988). Toward an instance theory of automatization. *Psychological Review*, *95*(4), 492.

Shields, B. J., Palermo, T. M., Powers, J. D., Fernandez, S. A., & Smith, G. A. (2005). The role of developmental and contextual factors in predicting children’s use of a visual analogue scale. *Children’s Health Care*, *34*(4), 273–287.

Shields, B. J., Palermo, T. M., Powers, J., Grewe, S., & Smith, G. (2003). Predictors of a child’s ability to use a visual analogue scale. *Child: Care, Health and Development*, *29*(4), 281–290.
